# Successful treatment of fusarium solani ecthyma gangrenosum in a patient affected by leukocyte adhesion deficiency type 1 with granulocytes transfusions

**DOI:** 10.1186/1471-5945-10-10

**Published:** 2010-10-07

**Authors:** Fethi Mellouli, Habib Ksouri, Ridha Barbouche, Mongi Maamer, Leila Ben Hamed, Slama Hmida, Assia Ben Hassen, Mohamed Béjaoui

**Affiliations:** 1 Service d'Immuno-Hématologie Pédiatrique. Centre National de Greffe de Moelle Osseuse. 2 rue Jebel Lakdhar, Tunis 1006. Tunisia; 2 Service des Laboratoires. Centre National de Greffe de Moelle Osseuse. 2 rue Jebel Lakdhar, Tunis 1006. Tunisia; 3 Laboratoire d'Immunologie cellulaire. Institut Pasteur de Tunis. Rue Charles Nicolle, Tunis 1002. Tunisia; 4 Laboratoire d'Hématologie. Centre National de Transfusion Sanguine. Rue Jebel Lakdhar, Tunis 1006. Tunisia

## Abstract

**Background:**

Ecthyma gangrenosum (EG) manifests as a skin lesion affecting patients suffering extreme neutropenia and is commonly associated with *Pseudomonas aeruginosa *in immunocompromised patients. Leukocyte adhesion deficiency I (LAD I) which count among primary immunodeficiency syndromes of the innate immunity, is an autosomal recessive disorder characterized in its severe phenotype by a complete defect in CD18 expression on neutrophils, delayed cord separation, chronic skin ulcers mainly due to recurrent bacterial and fungal infections, leucocytosis with high numbers of circulating neutrophils and an accumulation of abnormally low number of neutrophils at sites of infection.

**Case Presentation:**

We report at our knowledge the first case of a child affected by LAD-1, who experienced during her disease course a multi-bacterial and fungal EG lesion caused by *fusarium solani*. Despite targeted antibiotics and anti-fungi therapy, the lesion extended for as long as 18 months and only massive granulocytes pockets transfusions in association with G-CSF had the capacity to cure this lesion.

**Conclusion:**

We propose that granulocytes pockets transfusions will be beneficial to heal EG especially in severely immunocompromised patients.

## Background

Leukocyte adhesion deficiency I (LAD I) is an autosomal recessive disorder characterized by defect in CD18 expression on neutrophils, leading to leucocytosis with high numbers of circulating neutrophils and an accumulation of abnormally low number of neutrophils at sites of infection. Recurrent bacterial and fungal infections count among clinical complications of such disorder [1]. Nonetheless, ecthyma gangrenosum (EG) which represents a cutaneous infection mainly associated with pseudomonal sepsis in immunocompromised patient suffering extreme neutropenia [2,3], was never reported in the setting of LAD I.

We report a case of a child affected by LAD-1, who experienced a *fusarium solani *EG lesion. The lesion extended for as long as 18 months and only massive granulocytes pockets transfusions in association with G-CSF had the capacity to cure this lesion.

## Case presentation

A 9 years old girl affected by LAD-1 developed after a fall an abscess on her knee. Cultures taken from the lesion revealed a *Pseudomonas aeruginosa *infection. She was treated with amikacine 400 mg/day and imipenem 400 × 2 mg/day for three weeks and local disinfection. Lesion cultures performed one month later were negative. As all patients affected by such immunodeficiency, she was constantly under prophylactic treatment [25 mg/Kg/2 days (sulfamethoxazole + trimetoprime) and 10 mg/Kg/day Itraconazole].

Three months later, the patient was readmitted with 7 cm diameter erythematous and edematous lesion around necrotic center consistent with EG. The patient was apyretic and no pus was present. Laboratory findings were: peripheral WBC 23,000 mm3 (86% neutrophils); hemoglobin 8 g/dL. C-reactive protein was 106 mg/dL. Skin biopsies showed epidermal necrosis and ulceration with inflammatory granular cell infiltrates composed largely by lymphocytes, plasma cells, histiocytes, macrophages and rare eosinophiles. At that time, a necrotic vasculitis was confirmed but no bone lesion was found.

**Figure 1:**
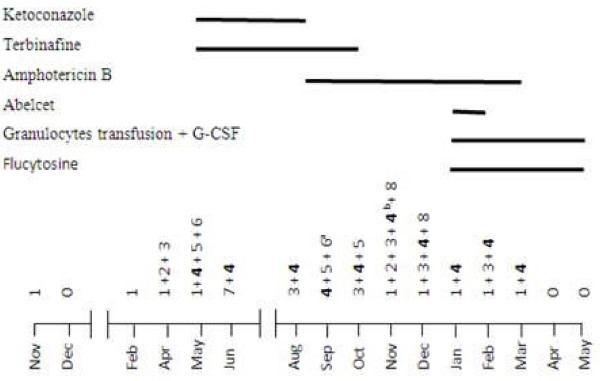
**Evolution of isolated bacteria in EG and blood cultures**. Along with bacteria identification, a targeted antibiotic therapy was administrated. G-CSF: Granulocytes-colony stimulating factor. **^a^**: blood culture; ^b^: blood and EG culture. 1- *Pseudomonas aeruginosa; *2- *Enterobactium cloacae; *3- *Enterobacterium faecalis; ***4**- *Fusarium solani; *5- *E. coli; *6- *Streptococus epidermidis; *7- *Staphylococus haemolyticus; *8- *Staphylococus epidermidis*

During 11 months and despite targeted antibiotic and anti-fungal therapy, cultures taken both from the lesion and from the blood were positive for several bacteria (especially *Pseudomonas aeruginosa*) and a persistent *fusarium solani*. While a Broviack central catheter for amphotericine B administration was installed, cultures remained positive and the lesion enlarged with none improvement (Figure 1). X-ray radiography showed bone lesions and signs of demineralization on the upper extremity of the shinbone. Bone scan revealed a periosteal reaction and magnetic resonance imagery a medullar edema.

In front of this over a year persistent infection, we started transfusions of irradiated, phenotyped granulocytes pockets (3 pockets per week in median) along with G-CSF 5 μg/Kg/day; liposomal amphotericine B (Abelcet 2 mg/Kg/day), amphotericine B (0,3 mg/Kg/day up to 2 mg/Kg/day), flucytosine, and targeted antibiotics. Amphotericine B was stopped after 6 months and only granulocytes transfusions, G-CSF and Flucytosine were administered for the 2 latter months (Figure 1). After almost 5 months of such treatment cultures became negative, EG reduced progressively and then disappeared completely. Healing was complete but poorly formed as for patients suffering of such primary immunodeficiency.

## Discussion

EG is one of the most serious cutaneous infections which occurs frequently in patients with neutropenia and underlying malignancies [4,5]. According to its pathogenesis, there are two main types of EG, with and without bacteremia. Skin lesions of the bacteremic type occur by hematological spread and blood cultures are positive, whereas in the better prognosis nonbacteremic form, lesions occur on the skin where organisms are inoculated [6]. Even though most lesions are located in the gluteal and perineal regions [7], our patient had a knee lesion following a trauma. After a restricted skin location during months, there was a germs blood spread and bacteremia episodes that may be responsible of EG persistence in our patient. Moreover, lack of neutrophils migration to infectious sites because of LAD constitutes somewhat a delay of treatment and may explain secondary bacteremia as reported [8].

*Pseudomonas aeruginosa *was the first bacteria isolated and was almost always present [9] with *fusarium solani *in our patients' lesion (Figure 1). Less commonly, non-pseudomonal pathogens like *Citrobacter freundii*, *E. coli*, *Aeromonas hydrophila*, *Stenotrophomonas maltophilia*, *Staphylococcus aureus*, and non-bacteremic states have been associated with EG [2]. Only one case of *fusarium solani *EG about a child affected by acute leukemia relapse was published [10]. Evolution was favorable and the child was completely cured when he recovered from aplasia. The fundamental difference between our case and those reported is the permanent condition of granulocyte inefficiency for our patient. Whereas for patients not affected by LAD I, treatment result can't be completely dissociated of granulocyte recovery. While neutropenia represent [2,3] the major predisposing factor for EG, the failure of neutophils to reach lesion location due to adhesion molecule deficiency in our patient, confirms the prominent role of these cells in curing such lesion. The therapeutic strategy of giving G-CSF beside transfusions was used to extend granulocytes half life. G-CSF was yet used in a child with acute lymphoblastic leukemia suffering from disseminated hyalohyphomycosis due to *fusarium solani *who developed an ecthyma gangrenosum-like lesion [11]. This particular therapy supplies fresh normal granulocytes able to supplement drug effects. As granulogytes deficiency and/or inefficiency constitutes the *primum movens *of EG occurrence, granulocytes transfusions along with targeted drug therapy seems to be the best supportive mean to cure EG lesions, especially in severely immunocompromised patients.

For our patient, EG lesion was a result of a relatively unusual germ (*fusarium solani*) and extended over one year despite aggressive and specific therapy. Bacterial and *fusarium solani *persistence represented an aggravating element either by itself or by the massive antibiotic therapy used. Both intensive monitoring and targeted therapy probably with the overriding granulocytes transfusion role permitted EG healing.

An oral informed consent was obtained from the father of the patient for publication of this case report.

## Conclusion

EG is a rare dermatologic infection due generally to bacteria and is commonly observed in patients with malignancy or neutropenia. Occurrence of such lesion secondary to *fusarium solani *in LAD I affected patient represents to our knowledge the first reported case. Granulocytes transfusions seem to be a good therapeutic option in such patient. This therapy can be generalized in severely immunocompromised patients affected by EG and/or in worst prognosis EG septicemic forms.

## Competing interests

The authors declare that they have no competing interests.

## Authors' contributions

FM: Participated in clinical follow-up and in writing the manuscript.

HK: Participated in biological follow-up and in writing the manuscript.

RB: Carried out the immunoassays.

MM: Participated in blood phenotyping for patient transfusions.

LBH: Participated in blood phenotyping for patient transfusions.

SH: Participated in blood phenotyping for patient transfusions.

ABH: Participated in bacteria and fungi identification.

MB: participated in clinical follow-up and in writing the manuscript.

All authors read and approved the final manuscript.

## Pre-publication history

The pre-publication history for this paper can be accessed here:

http://www.biomedcentral.com/1471-5945/10/10/prepub
